# Impacts of international sanctions on Iranian pharmaceutical market

**DOI:** 10.1186/2008-2231-21-64

**Published:** 2013-07-31

**Authors:** Abdol Majid Cheraghali

**Affiliations:** 1 Department of Pharmacology, University of Baqiyatallah Medical Sciences, Tehran, Iran

**Keywords:** International sanctions, Medicines, Iran

## Abstract

Iran in recent decade faced several regional and international sanctions in foreign trade, financial and banking services. Iran national pharmaceutical industry has always played a major role in providing medicines to the Iranian patients. However, following the sanctions it has faced profound difficulties for importing of both finished products and pharmaceutical raw materials. Although medicines are exempted from sanctions, due to restriction on money transaction and proper insurance Iranian pharmaceutical companies have to pay cash in advance for imports of medicines and raw materials or to secure offshore funds at very high risks. Current situation in Iran pharmaceutical market confirms that the sanctions against Iran are affecting ordinary citizens and national health sector which resulted to reduction of availability of lifesaving medicines in the local market and has caused increasing pain and suffering for Iranian patients.

## Background

In the past decades, regional and international economical sanctions have been used by some countries in order to impose political intentions in the sanctioned countries. The main objective of sanctions is to interrupt a country’s diplomatic and/or economic relations in a worldwide scale. Sanctions could be categorized based on its scope on a regional or international base. Local or regional sanctions are usually employed by some limited countries against one specific country. This obviously is less effective for reaching to its objectives since the sanctioned country usually diverts its political and economical relations to other countries. Article 41 of the United Nation (UN) Charter is usually referred to for imposing international sanctions against UN member states. This article authorizes international community to implement measures not involving the use of armed forces including complete or partial interruption of economic relations for a specific objective.

In the past decades, most of the time, sanctions have been employed by developed market economies against weaker and more dependent states. Regional or international sanctions have been used in recent decades against countries such as Haiti, Iraq, Yugoslavia, South Africa, Cuba, Burundi, Nicaragua, Gaza Strip, Zimbabwe and Iran [1-4]. However with exception of South Africa there is not any evidence indicating that sanctions have reached their primarily objectives as it was claimed. Sanctions imposed by European Union (EU) against Zimbabwe are a clear example that sanctions most of the time fail to reach their originally specified targets. As a contingency measure against EU sanctions Zimbabwe shifted its focus from the EU and its western allies and formed economic partnerships with the Eastern countries [2].

Although most of the time it is claimed that sanctions and especially “targeted sanctions” aimed political leadership of sanctioned countries, unfortunately sanctions are blunt instrument which could hurt large number of people. There is now substantial evidence that regional and international sanctions cause severe civilian hardship and profound social and economic dilemma for ordinary people of the sanctioned country. Sanctions could devastate countries economical infrastructure in different sections and most notably in health care sector. Sanctions could profoundly disrupt health services in the sanctioned country. The impact of sanctions on health and health services is not limited to difficulties with supply of medicine and will go far beyond to disrupt health services.

Although almost all sanctions in the recent decades had provision for exemptions of medicines and food stuffs, sanctions through complications in transportation, difficulty in transferring hard currencies or either lack of capital commonly lead to disruption of health services and even basic nutrition of the ordinary people in the sanctioned countries. Published reports show that economical sanctions will increase suffering and death among civilians particularly among the most disadvantaged and vulnerable groups including mothers, children and patients with chronic disease. Obvious reduction in public resources allocated for heath sector along with restrictions on importation of vital medicines and equipments will ultimately result to a weakened physical and medical infrastructures and strain the ability of health system to provide medicines and services to the patients. Sanctions are associated with reported substantial declines in health and welfare of the ordinary citizens of Cuba, Iraq, former Yugoslavia, Gaza Strip, Burundi, Zimbabwe and Nicaragua [1-4]. Although countries such as Cuba have managed to establish a viable health care service for their citizens despite decades long unilateral sanctions by USA, others may not be successful as such. Iraq for example had invested heavily in health and education services in the 15 years prior to the embargo and in 1990 it had an advanced health care system. However, the county lost all of its achievements in health sector following international sanctions [1]. These changes left all Iraqis at greater risk of poor health outcomes just before military invasion by USA and its allies.

In related to its nuclear activities, Iran in recent decade faced several regional and international sanctions. In addition to international sanctions, EU and USA have also imposed restrictions on cooperation with Iran in foreign trade, financial and banking services, energy sectors and technologies, and banned the provision of insurance by insurers in member states to Iranian-owned companies. Before sanctions Iran national pharmaceutical industry played a major role in providing essential medicines to the Iranian patients [5]. In recent years the country’s national industry was also able to locally manufacture several lifesaving biopharmaceuticals [6]. However, following the sanctions Iran pharmaceutical market has faced profound difficulties for importing of both finished products and active pharmaceutical ingredient (API) for fulfilling its responsibility on providing necessary medicines for the patients in need. In the other hand as a result of sanctions and due to reduction of the country’s international incomes, Iran national currency (Rial) has also experienced drastic devaluation against international currencies. These have caused both substantial price increase and shortage of the medicines in Iran pharmaceutical market. This obviously has imposed unnecessary pain and suffering on Iranian patients and their families.

Although medicines are always exempted from sanctions, many international companies failed to fill orders from Iran due to restriction on money transaction, proper insurance and sometimes assurances that the item indeed was exempted from the embargo. In the other hand local pharmaceutical companies are finding it extremely difficult to access lines of credit for importing medicines or APIs. Currently Iranian pharmaceutical companies have to pay cash in advance for imports of medicines and APIs or to secure offshore funds at very high risks if they could do it at all.

In order to improve availability and affordability of plasma derived medicines (PDM) since 2005 Iran has successfully implemented a contract fractionation program for surplus of recovered plasma produced in its national transfusion service. In this program locally produced plasma by Iran Blood Transfusion Organization (IBTO) is sent to the fractionator and end products are returned to the country for distribution into the local market [7]. The medicines received under this contract fractionation program provide for 100% of IVIG and clotting factor IX used in Iran, as well as 15% of the clotting factor VIII and 40% of the albumin. The cost differential between imported products and those obtained via the contract fractionation program resulted in significant saving for Iran health sector. Therefore Iran’s contract fractionation program has substantially improved accessibility and affordability of PDM and resource allocation in the national health system [8].

However, unfortunately in past two years, economical restrictions imposed by EU on Iran banking system and flow of the money has pushed Iran national contract fractionation program to the verge of total collapse. These restrictions on importation of PDM into Iran have also created a dreadful condition for patients in need of such medicines including hemophilia and primary immunodeficiency disorders patients who need these medicines for their survival. Unavoidably price increase and more importantly shortage of these medicines in the market have drastically compromised treatment of these patients. Although establishment of a contingency plan might enable IBTO to continue its contribution for providing lifesaving PDM for these patients, continued pressures and restrictions by EU on Iran health sector might get its high toll in near future. One practical contingency plan could shift IBTO to search for partners in non European countries. However, due to limited number of qualified plasma fractionators in non European countries e.g. Asian and South America, this approach to the East might not be fruitful approach to survive the contract fractionation program at least in short term.

## Conclusion

There is now consensus among political scientists that the record of sanctions in achieving their stated objectives is very low [1]. Instead ordinary people who live in sanctioned countries have to bear costs attributed to the sanction. Although most published reports of assessment of sanctions effects on health focuses on clinical health services, shortages of medicines and inability to diagnose or treat illness and the functional loss of equipment due to lack of access to spare parts is common.

Results of observations from current situation in Iran pharmaceutical market confirm that the sanctions against Iran are affecting ordinary citizens and health sector which resulted to reduction of availability of lifesaving medicines in the local market. As the Iran’s political leadership has withstood the economic sanctions over last decade, it is clear that sanctions have not achieved their stated political to objectives. However, this is obvious that sanctions against Iran, due to lack of timely access to the lifesaving medicines, has caused increasing pain and suffering for Iranian patients.
